# Translating Preclinical Research for Exercise Oncology: Take It to the VO_2max_

**DOI:** 10.3389/fonc.2020.575657

**Published:** 2020-10-02

**Authors:** Donald M. Lamkin, Theodore Garland

**Affiliations:** ^1^Norman Cousins Center for PNI, Semel Institute for Neuroscience & Human Behavior, University of California, Los Angeles, CA, United States; ^2^Department of Psychiatry & Biobehavioral Sciences, David Geffen School of Medicine at UCLA, Los Angeles, CA, United States; ^3^Jonsson Comprehensive Cancer Center, University of California, Los Angeles, CA, United States; ^4^Department of Evolution, Ecology, and Organismal Biology, University of California, Riverside, CA, United States

**Keywords:** breast cancer, exercise dose, exercise oncology, physical activity, preclinical research, translational science, tumor hypoxia, VO_2max_

## Abstract

Several observational studies have found that the risk for breast cancer is significantly reduced in persons who engage in greater amounts of physical activity. Additional observational studies of breast cancer survivors indicate that greater physical activity before or after diagnosis associates with reduced disease-specific mortality. However, no large randomized controlled trials have examined the effect of structured exercise training on disease outcomes in breast cancer. Among the many hurdles in designing such trials lies the challenge of determining how a given regimen of exercise from efficacious preclinical studies can be extrapolated to an equivalent “dose” in humans to guide decisions around treatment regimen in early-phase studies. We argue that preclinical researchers in exercise oncology could better facilitate this endeavor by routinely measuring changes in exercise capacity in the subjects of their breast cancer models. VO_2max_, the maximal rate of whole-organism oxygen consumption during a progressive exercise test, is emphasized here because it has become a standard measure of cardiorespiratory fitness, is well-integrated in clinical settings, and scales allometrically among nonhuman animals in preclinical research and breast cancer patients/survivors in the clinic. We also conduct secondary analyses of existing whole-transcriptome datasets to highlight how greater uptake and delivery of oxygen during exercise may reverse the typically hypoxic microenvironment of breast tumors, which often associates with more aggressive disease and worse prognosis.

## Introduction

Over the past 30 years, exercise therapists and other clinical investigators have conducted randomized controlled trials (RCTs) with cancer patients to examine the effect of structured exercise training on a variety of dependent variables. Although breast cancer has received the most attention, other cancer types studied include prostate, lung, colorectal, gastrointestinal, gynecologic, testicular, bladder, lymphoma, leukemia, and brain (1). The first such RCT was conducted in the late 1980s and found that a structured exercise intervention involving thrice-weekly supervised aerobic activity with breast cancer patients significantly reduced treatment- related nausea, reduced body fat, and increased physical fitness (2–4). However, spurred by emerging observational evidence that physically active women had a lower risk of developing breast cancer, clinical investigators soon began conducting RCTs to examine the effect of exercise on biomarkers that associate with breast cancer prognosis (e.g., serum insulin, C-reactive protein, immune cell activity), doing so during and/or after initial adjuvant treatment (i.e., radiotherapy and/or chemotherapy) (5, 6). Although informative, such studies have not led to any firm conclusions about the ability of structured exercise training to modulate disease outcomes in breast cancer patients (i.e., disease-free survival, cancer-specific mortality, overall mortality). More recently, a “window of opportunity” RCT examined the effect of pre-surgery exercise training on specific tumor biology parameters in excised tumors (e.g., cell proliferation, apoptosis) (7). Although informative and quite novel in its collection of pre- and post-exercise whole-genome gene expression (8), again, this study was unable to provide any firm conclusions about the ability of structured exercise training to alter the course of breast cancer disease.

Definitive conclusions about any treatment effect come from the successful completion of large phase III RCTs (9). However, as outlined by those working in exercise oncology, the path that leads to successful design of such trials contains several unique obstacles for those who wish to determine the effects of a non-pharmacological treatment, such as exercise, on cancer outcomes (10, 11). Among those obstacles lies the challenge of determining how a given regimen of exercise from preclinical studies, which may have been found to be efficacious at inhibiting a given type of tumor, can be extrapolated to an equivalent “dose” in humans to guide decisions around treatment regimen in early phase I/II studies. In conventional drug development, investigators may rely on several established approaches (derived from pharmacokinetics) to estimate an efficacious dose for humans from doses found to work in animals (12, 13). These approaches aim to achieve a pharmacodynamic response in humans that is similar to what was measured in prior supportive animal studies by accounting for known interspecies differences in the pharmacokinetics of the drug class (e.g., absorption, distribution, metabolism, excretion) and applying appropriate scaling factors related to differences in body size (12, 13). In contrast, for a behavioral treatment like exercise, such pharmacological approaches do not seem to readily apply.

Interspecies scaling factors utilized in conventional drug development have their origin in a long history of studies that have empirically determined allometric scaling equations for translating several normal physiological parameters between nonhuman animals and humans. Moreover, changes in such physiological parameters likely associate with exercise's mechanism(s) of action on cancer inhibition in both animal models and humans alike (14). Among the several physiological parameters known to associate with exercise is resting heart rate (HR), which varies allometrically with body mass (BM) among land-dwelling mammals as HR = 212 ∙ BM^−0.22^ beats per min (BM in kg) (15). During restful periods, the operation of HR in conjunction with other parameters—including cardiac output and respiratory minute volume—results in approximately the same percentage of oxygen being extracted from ventilated air and delivered to the body regardless of the mammal's size (16). However, the dynamics surrounding oxygen consumption change during exercise, and the overall result can be measured by another physiological parameter—the maximal rate of whole-organism oxygen consumption during a progressive exercise test (VO_2max_).

## Translating Exercise Dose: Focus on the Response

VO_2max_, the maximal rate of whole-organism oxygen consumption (typically over 1 min) during a progressive exercise test, has become the standard measure of cardiorespiratory fitness and a robust indicator of exposure to routine aerobic/endurance activity (17). Investigators have long utilized VO_2max_ to investigate the effect of exercise training on cardiorespiratory fitness and other risk factors for cardiovascular disease (CVD) [e.g., (18)]. Not surprisingly, exercise oncologists have also commonly used this measure (or the related VO_2peak_) to determine the effects of structured exercise interventions in numerous RCTs. A recent meta-analysis of 48 such RCTs (the most prevalent being for breast cancer) found that VO_2peak_ in cancer patients was significantly increased by exercise therapy in comparison to cancer patients in the control group (1), consistent with many other studies of unaffected individuals (19).

Given that it serves as a robust indicator of exposure to routine aerobic/endurance activity (19), pre–post changes in measures of cardiorespiratory fitness like VO_2max_ have been thought of as the “pharmacokinetic equivalent” for an exercise trial or as a manipulation check wherein the exercise regimen is shown to have done its job (8). This is prudent because it is becoming increasingly clear that a given regimen of exercise does not induce the same training effect across all subjects who equally complete the same regimen (be they human or animal model) (20). Individual variations in both baseline and acquired VO_2max_ following exercise exposure have a substantial genetic component (21). However, this issue is not unique to VO_2max_, as several other measures of training effect show heterogeneity [e.g., running capacity, submaximal heart rate, submaximal systolic blood pressure, fasting high-density lipoprotein (HDL) cholesterol levels] (20, 22). Thus, given the supposition that global physiological alteration from greater physical activity contains the likely mediator(s) between exercise and cancer inhibition, perhaps it is less important to attempt extrapolation of the exercise regimen *per se* from preclinical studies and more important to extrapolate the measure of global physiological alteration.

Preclinical research in exercise oncology could better inform clinical investigation if the former began to routinely measure pre- and post-intervention exercise capacity. VO_2max_ is emphasized here because, as noted above, it is a gold standard and has become well-integrated into clinical settings, including oncology. Exercise physiologists and clinicians alike note the power of VO_2max_ to noninvasively and objectively determine the efficacy of aerobic, endurance-exercise training programs in clinical settings (23, 24). If more preclinical research in the exercise oncology realm did the same, then such exercise data could be analyzed in relation to a plethora of mechanistic tumor biology data that can be captured in a well-controlled preclinical experiment. More pertinent to the point at hand, the connections that are discovered with VO_2max_ could be more readily translated for consideration at the clinical level, as VO_2max_ cuts across all orders of the class Mammalia (25).

As with the exercise-related physiological parameters noted above, VO_2max_ scales allometrically across mammals. The most recent large-scale empirical determination utilized phylogenetically informed statistics when analyzing a total of 77 species, including humans, and determined the relationship to be VO_2max_ = 0.303 ∙ BM^0.837^ ml/min (excepting bat, horse, and pronghorn, which have unusually high values) (BM in g) (25). The VO_2max_ of a 35-g adult mouse is predicted to be, on average, about 5.94 ml/min, which falls within the measured range for sedentary laboratory mice of this size [e.g., (26)]. But because of the fractional exponent for BM in this equation (i.e., as the species gets larger, VO_2max_ gets larger, though less than proportionately), the VO_2max_ of an average adult human (62 kg) is predicted to be around 3,109 ml/min, which also falls within the measured range for adult men and women in national reference standards (17).

Differences between animals in pre-intervention exercise capacity (i.e., baseline VO_2max_) or in the relative change at post-intervention (i.e., within-subject % increase in VO_2max_) could be used to test for association with tumor inhibition and/or mechanistic variables in the tumor microenvironment. For example, for one given cancer model with specific tumor biology features, it may be found that a certain *minimum amount* of exercise capacity, measured by VO_2max_ (i.e., a *threshold*), needs to be reached by the subject in order to achieve a monolithic inhibitory effect. Conversely, a different cancer model with a different tumor biology may show that the *relative increase* in VO_2max_ from baseline has tumor-inhibitory effects in a dose-response manner, or, after a certain threshold is achieved, the relative increase exerts greater amounts of tumor inhibition in a dose-response manner. In either case, the amount of VO_2max_ found to serve as a threshold in the efficacious preclinical study may be more translatable (and relevant) than the animal exercise regimen that was used to derive the VO_2max_. Suppose a given tumor model is found to exhibit a threshold VO_2max_ value that is 10% above the predicted average value for the animals in the study, given their BM. Such a finding may then suggest that future clinical investigations consider a phase I/II trial where cancer survivors (with similar residual tumor biology to that of the model) are exposed to a structured exercise regimen in the intervention arm that aims to increase (and maintain) a VO_2max_ that is 10% above the predicted average value for each person based on each person's specific BM.

## VO_2max_: Direct Effects on Tumor Biology?

Exercise-induced changes in cardiorespiratory fitness may not always correlate with other exercise-induced changes that might have more direct bearing for the tumor biology of a given model. Exercise oncologists, focusing on cancer treatment-induced cardiovascular toxicity, have pointed to research that finds slightly different exercise regimens can have essentially the same augmenting effect on peak oxygen consumption but differ in their salutary effects on plasma lipoproteins (27, 28). Similar divergence across different regimens for VO_2max_ and breast cancer risk covariates (e.g., insulin, adipocytokines, inflammatory proteins, etc.) (29) may also exist.

Notwithstanding possible dissociations with VO_2max_, it seems appropriate to note that the primary constituent of VO_2max_, i.e., *greater whole-body oxygen consumption*, may in itself have direct effects on breast tumor biology that become substantial in the course of increasing and maintaining a relatively higher VO_2max_. Tumors tend to be hypoxic microenvironments, and greater tumor hypoxia is associated with poorer response to radiation treatment, poorer response to chemotherapy, greater likelihood of metastasis, and worse survival (30–32). However, in preclinical models of breast cancer, voluntary wheel running has been found to reduce tumor hypoxia (33, 34). Thus, it would appear that, in the course of blood flow alteration and greater tissue oxygenation for working muscles during physical exercise (35), there is a collateral increase in oxygenation of mammary tissue and/or the tumors that inhabit this area.

Preclinical evidence suggests that tumor vessel normalization is a key mechanism by which exercise is able to reduce hypoxia and greatly improve the antitumor effectiveness of chemotherapy in breast cancer (33). Such evidence is consistent with other research that finds tumor vessel normalization with the drug bevacizumab can improve the direct antitumor effect of chemotherapy in both early- and late-stage breast cancer (36, 37). However, the effect of bevacizumab is short-lived as drug resistance develops (38), whereas the effect of exercise may endure (33), at least if the exercise regimen is maintained. We emphasize, though, that there has been no direct comparison of exercise vs. bevacizumab on tumor vessel normalization and the effects of antitumor chemotherapy.

Other research suggests that normalizing the tumor vasculature and increasing tissue oxygenation may improve immunosurveillance and immunotherapy (39, 40). Given the less than ideal response of breast cancer to checkpoint blockade immunotherapy, which antagonizes negative feedback signaling to antitumor immune cells, investigators have speculated that greater benefit may be achieved by combining such checkpoint blockade drugs with other treatments (41). To this end, it is argued that the next generation of preclinical exercise studies in cancer should evaluate the interaction between exercise and novel immunotherapies (42). Breast cancer has long been thought of as a non-immunogenic or “immunologically cold” malignancy (43), which may be due, in part, to the high propensity of myeloid cells in breast tumors to exhibit an immunosuppressive role (44). However, emerging evidence shows that such cells may become less immunosuppressive in tissue environments that are less hypoxic as a result of greater physical activity [reviewed in (14, 45)].

**Figure 1:**
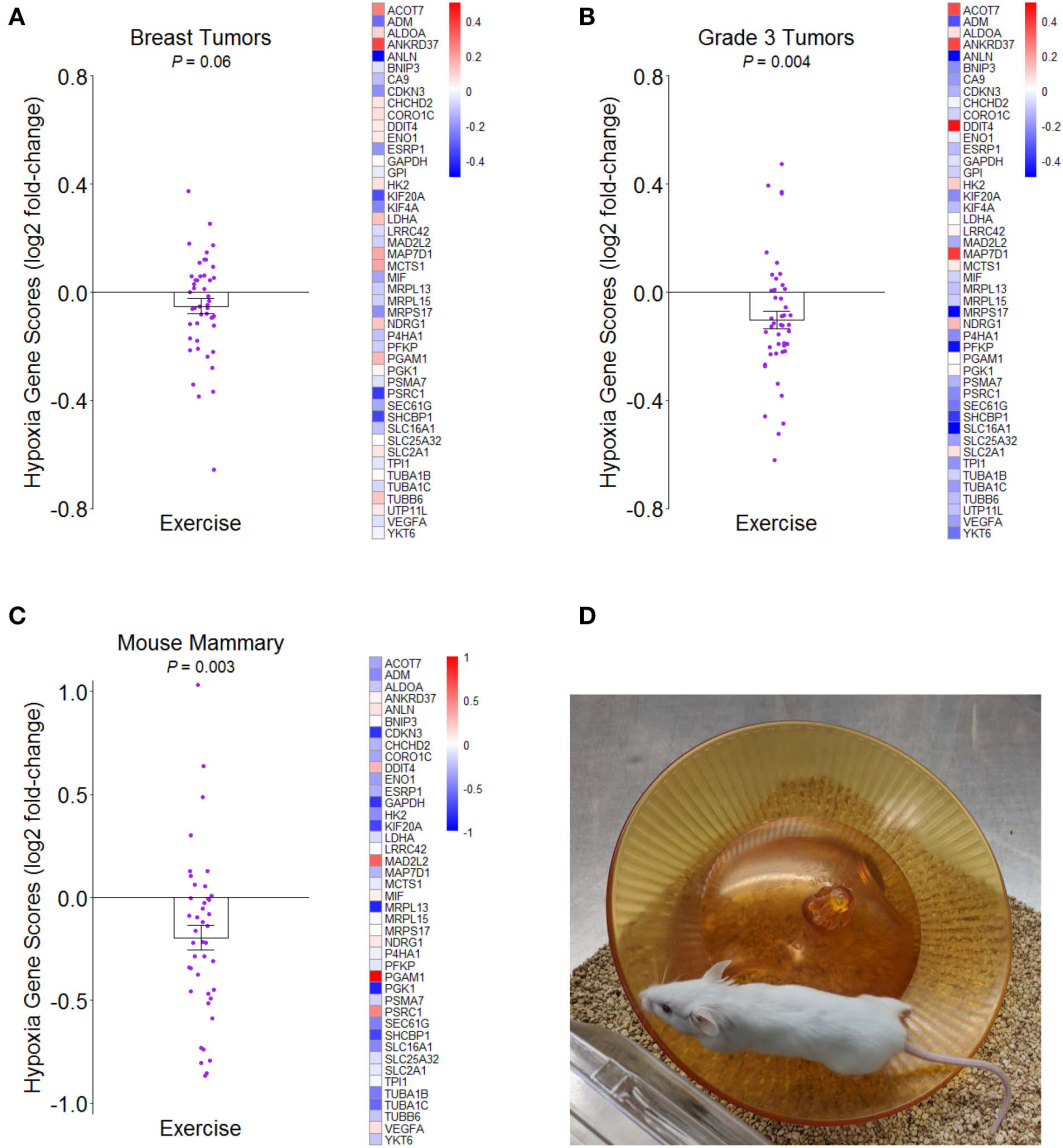
Effects of greater routine physical activity on hypoxia in mammary tissue. **(A)** Mean ± SEM fold-change for hypoxia genes from tumors of breast cancer patients randomized to exercise vs. control conditions in Ligibel et al. (7) on log2 scale. Reference hypoxia gene set from Buffa et al. (46). Distinct data points given for each gene in plot. Heatmap of mean fold-changes for distinct hypoxia genes in exercise group vs. control group on log2 scale. *P*-value indicates significance of difference between groups in mean change scores from transcriptome representation analysis (TRA). **(B)** Mean ± SEM and heatmap as in **(A)** but for breast cancer patients with grade 3 tumors. **(C)** Mean ± SEM fold-change for same reference hypoxia gene set in mammary tissues of mice randomized to exercise vs. control in NCBI GEO accession number GSE150620 on log2 scale. Heatmap of mean fold-changes for distinct hypoxia genes in exercise vs. control group on log2 scale. TRA *P*-value indicates significance of difference between groups in mean expression scores of genes. **(D)** Representative image of activity paradigm for mice in **(C)**.

We examined the effect of a structured exercise regimen for breast cancer patients on tumor hypoxia by analyzing whole transcriptome data from the innovative RCT by Ligibel et al. (7) (NCBI GEO accession number GSE129508). Patients randomized to the exercise condition completed two 60–90-min trainer-supervised exercise sessions per week over a median period of about 4 weeks and completed additional aerobic exercise on their own between supervised sessions with a pedometer to track activity amounts. Using a hypoxia gene expression signature that was developed in part from analysis of several breast cancer studies (46) (see Supplementary Data File 1), transcriptome representation analysis (TRA) (47) indicated that pre- to post-intervention change in hypoxia was more reduced in tumors of exercising patients than in the tumors of control patients, though the effect was marginal (*P* = 0.06) (Figure 1A). Given the meta-analytic finding that high tumor grade significantly correlates with tumor hypoxia in breast cancer patients (32), we analyzed patients with low-grade (1/2) and high-grade (3) tumors separately and found that the effect of exercise on hypoxia was significantly stronger for patients with high-grade tumor (*P* = 0.004) (Figure 1B).

We then determined whether this same hypoxia gene expression signature would indicate less hypoxia in the mammary tissues of mice after being randomized to voluntary wheel running for 2 weeks prior to mammary cancer cell engraftment (NCBI GEO accession number GSE150620). Compared to sedentary control mice that also received mammary cancer, wheel-running mice had less hypoxia gene expression (*P* = 0.003) (Figures 1C,D). Together, the results suggest that greater routine physical activity can reduce hypoxia in breast tumors and surrounding mammary tissue in both humans and mice. Although VO_2max_ was not measured in either of these studies, we speculate that it would likely correlate with tissue hypoxia in an inverse manner.

## Conclusions

The evidence of a beneficial link between physical activity and breast cancer has been around for a long time. Thus, it may be somewhat surprising that a large phase III RCT for the effect of structured exercise training on disease outcomes has not been launched. By now, multiple observational studies have found that the risk for breast cancer is significantly reduced in persons who engage in greater amounts of physical activity (48), and several more observational studies of breast cancer survivors indicate that greater physical activity either before or after diagnosis associates with reduced disease-specific mortality (49, 50). Likewise, meta-analysis of preclinical exercise studies in rodent models of breast cancer (i.e., mice and rats) finds an overall inhibitory effect in this cancer type (51), even though not all preclinical exercise studies in rodents have shown efficacy across all tumor types examined thus far [see review by (42)]. Thus, it is important to note that exercise may not be as beneficial for other cancer types. Nevertheless, given the enormity of the epidemiological findings for breast cancer, the World Health Organization's IARC (International Agency for Research on Cancer) has assigned its strongest evidence designation to physical activity as a preventative factor in breast cancer (52).

However, as noted at the beginning of this Perspective article, we face several challenges to determining the effects of a non-pharmacological treatment like exercise on cancer outcomes in the clinical setting. We have focused specifically on one—the challenge of extrapolating the overall exercise dose from efficacious preclinical studies to help inform clinical treatment regimens. Currently, the basic exercise dose that is recommended for cancer survivors largely follows general physical activity guidelines for adults with chronic conditions, which aims for at least 150 min per week of aerobic activity, with two or more days per week of resistance training (53). Although VO_2max_ may be an imperfect indicator of exercise exposure (given the heterogeneity of all physiological training effects noted above, with likely genetic contributions), we contend that its reliability, validity, and translatability make its use a highly worthy endeavor. We also think that greater use of an objective measure of exercise exposure at the preclinical level will facilitate identification of the *essential biology* that is at work in the effect between physical activity and cancer inhibition (for those cancer types that are responsive to exercise). Exercise oncologists presciently understand that this is another challenge facing successful clinical trial development for the effect of structured exercise training on cancer outcomes. Most large definitive trials of “nonregulated” therapies for cancer (i.e., non-pharmaceutical) have failed to show any benefit, which may likely be due to a lack of prior studies (preclinical or otherwise) successfully identifying doses and scheduling that effectively altered the relevant underlying biology (11). Thus, investigators in exercise oncology do not want to go down that same fruitless path.

To conduct a rational, optimal trial design for a given tumor type, clinical investigators will benefit from knowing *what* downstream biological endpoint(s) to aim for with a structured exercise regimen. They will also benefit from knowing *how much* downstream biological endpoint is needed to induce inhibition of the given tumor type and, then, *how much exercise* is needed to bring about an efficacious amount of the downstream biological endpoint. Preclinical researchers can make great contributions to this venture by measuring the effect of exercise on tumor biology in their cancer models. They can make *greater* contributions by also measuring the dose of exercise in their cancer models in a way that is reliable, valid, and translatable.

## Data Availability Statement

The datasets presented in this study can be found in online repositories. The names of the repository/repositories and accession number(s) can be found at: https://www.ncbi.nlm.nih.gov/ (GSE150620, GSE129508).

## Ethics Statement

The studies involving human participants were reviewed and approved by Institutional Review Board, Dana-Farber/Harvard Cancer Center. The patients/participants provided their written informed consent to participate in this study. The animal study was reviewed and approved by Institutional Animal Use and Care Committee, UCLA.

## Author Contributions

DL wrote the initial draft of the article, in consultation with TG. DL conducted secondary analyses on existing datasets and constructed accompanying figures for the article. TG made critical revisions to the manuscript. Both authors contributed to manuscript revision and read and approved the submitted version. All authors contributed to the article and approved the submitted version.

## Conflict of Interest

The authors declare that the research was conducted in the absence of any commercial or financial relationships that could be construed as a potential conflict of interest.
